# Adherence to a Mediterranean-Style Diet and Effects on Cognition in Adults: A Qualitative Evaluation and Systematic Review of Longitudinal and Prospective Trials

**DOI:** 10.3389/fnut.2016.00022

**Published:** 2016-07-22

**Authors:** Roy J. Hardman, Greg Kennedy, Helen Macpherson, Andrew B. Scholey, Andrew Pipingas

**Affiliations:** ^1^Centre for Human Psychopharmacology, Swinburne University of Technology, Melbourne, VIC, Australia; ^2^Centre for Physical Activity and Nutrition Research, Deakin University, Melbourne, VIC, Australia

**Keywords:** nutrition, cognition, Mediterranean diet, clinical trials

## Abstract

The Mediterranean-style diet (MedDiet) involves substantial intake of fruits, vegetables, and fish, and a lower consumption of dairy, red meat, and sugars. Over the past 15 years, much empirical evidence supports the suggestion that a MedDiet may be beneficial with respect to reducing the incidence of cardiovascular disease, cancer, metabolic syndrome, and dementia. A number of cross-sectional studies that have examined the impact of MedDiet on cognition have yielded largely positive results. The objective of this review is to evaluate longitudinal and prospective trials to gain an understanding of how a MedDiet may impact cognitive processes over time. The included studies were aimed at improving cognition or minimizing of cognitive decline. Studies reviewed included assessments of dietary status using either a food frequency questionnaire or a food diary assessment. Eighteen articles meeting our inclusion criteria were subjected to systematic review. These revealed that higher adherence to a MedDiet is associated with slower rates of cognitive decline, reduced conversion to Alzheimer’s disease, and improvements in cognitive function. The specific cognitive domains that were found to benefit with improved Mediterranean Diet Score were memory (delayed recognition, long-term, and working memory), executive function, and visual constructs. The current review has also considered a number of methodological issues in making recommendations for future research. The utilization of a dietary pattern, such as the MedDiet, will be essential as part of the armamentarium to maintain quality of life and reduce the potential social and economic burden of dementia.

## Neurocognitive Aging and the Potential Associated Risk Factors

An aging population raises the potential for increased incidence of cognitive impairment (1). Preservation of brain function and the reduction of risk of neurological disorders have become key issues for society. In addition to the need to find treatments for disorders, such as Alzheimer’s disease (AD), it is becoming increasingly apparent that early intervention is critical for the maintenance of brain health across the life span and reducing the risk of accelerated neurocognitive decline (2). There is mounting evidence that risk factors for cardiovascular disease, stroke, diabetes, and other later-life chronic health conditions also exacerbate age-associated cognitive decline, which can lead to AD (3). Research is increasingly focusing on understanding how interventions, such as improving nutritional status and modifying risk factors that may impinge directly and/or indirectly on brain functioning, can reduce the risk of neurocognitive impairment (4). Closer adherence to a traditional Mediterranean-style diet (MedDiet) may be beneficial and protective with respect to addressing such risk factors, ultimately reducing the rate of cognitive decline (5).

Neurocognitive decline across the life span is well documented. In particular, cognitive faculties that involve speed of processing and memory steadily decline from the third decade of life (6). For example, the speed of retrieval of previously learned spatial locations from short-term memory is reduced by around 50% from the third to the eighth decade of life (7). While this slowing of memory retrieval is observed in all individuals, there is considerable intersubject variability in cognitive trajectories across the life span, particularly in later life (8). Individuals who are still cognitively intact, but who have shown accelerated cognitive decline, are potentially at risk of dementia and AD (9). It has been suggested that our individual cognitive trajectories relate to our overall health, and, as such, are potentially modifiable (10).

Aging affects multiple biological systems and organs, including the brain (11). Much research has focused on the investigation of gross neuroanatomical changes with age. In recent years, this area has advanced through the adoption of high-resolution volumetric techniques, such as magnetic resonance imaging (MRI). Such methods reveal brain volume reduction at a rate of 5% per decade after the age of 40 years, with the rate of reduction possibly increasing after the age of 70 years (12). Other areas of investigation include structural and functional changes at the cellular level. Neurocognitive aging is related to a lifelong incidence of molecular damage, with progressive destruction of both biomolecular and cellular components (6). There are also neurochemical changes. For example, levels of brain-derived neurotrophic factor (BDNF) are known to reduce, which can impact synaptic plasticity and neurogenesis in the aging adult brain (13). Amyloid plaques and neurofibrillary tangles, the hallmarks of AD, have been extensively researched and occur commonly with age in cognitively intact individuals (12). Given the relevance of these neuropathological changes to AD, the origin of these changes and the potential risk for accelerated neurocognitive decline is being widely researched (14, 15).

The cardiovascular system offers prime examples of the relationship between lifestyle, health, and aging. As we age, our arteries stiffen, and there is increased risk of cardiovascular disease, which has been associated with poor diet and lack of exercise (16). Stiffening of the arteries and increased blood pressure also increases the risk of stroke and neurodegeneration. Elevated systolic blood pressure has been shown to increase gray matter volume loss, which may eventually result in cognitive dysfunction (17). In addition to the consideration of increasing cardiovascular risks, it is important to also consider the important relationship between blood pressure, high cholesterol, and obesity (3). These three factors are associated with what is referred to as the metabolic syndrome, a cluster of conditions typically including increased blood pressure and elevated blood sugar levels that can be associated with excess body fat around the waist and abnormal cholesterol levels. When combined, these conditions may increase the risk of heart disease, stroke, and diabetes (18).

Investigation of physiological changes at the molecular level has helped to elucidate possible mechanisms of action for the aforementioned changes in neural and cardiovascular functioning. Inflammatory processes often increase with age and contribute to chronic disorders, such as arthritis, diabetes, and cardiovascular disease, as well as to neurodegenerative disorders, such as AD (19). It has also been proposed that age is associated with increased oxidative stress and free radical damage (20). Inflammation and oxidative stress may therefore be critical targets for the amelioration of declining health and brain function across the life span, which can potentially be addressed through improved nutrition and increased physical activity (21). However, as discussed below, there are very few controlled clinical trials that have utilized these simple interventions to investigate their effects on cognition in older individuals.

## Nutrition and Cognitive Status

The variability between individuals’ cognitive abilities in later life has been well documented (11, 12, 22, 23). The source of this variability has been related to both non-modifiable factors, such as age, gender, and genetics, as well as modifiable risk factors, such as stress, education, psychological well-being, and exercise (24).

Another particularly important modifiable risk factor for cognitive decline is dietary status. There is a wealth of literature, from both animal studies and human health research, indicating that diet can exert profound effects on biological aging (25–35). Diet can also affect other risk factors, such as inflammation and oxidation. Diets that are low in energy and that act to reduce oxidative stress may be protective against cognitive decline (25, 36). Conversely, a diet that is high in energy and acts to increase oxidative stress may be considered a risk factor for impaired cognitive functioning (37).

Additionally, there are neurotrophic and neuroendocrine factors that may play a role in the cognitive responses that relate to food intake (38). Increasing consumption of fish and seafood by as little as one portion per month may be protective against stroke (39). Along with a healthy diet, there is evidence that low or moderate intake of alcohol may reduce cardiovascular and neurocognitive risk, with heavy drinkers tending to suffer more coronary infarcts and dementia (40, 41).

The dietary B-complex vitamins, which include B1 (thiamine), B2 (riboflavin), B3 (niacin), B5 (pantothenate), B6 (biotin, folate), and B12 (cobalamin), are important regulators of neurotransmitter function (42). Vitamin B6, in particular, is an important cofactor for the enzymes that synthesize the neurotransmitters serotonin, epinephrine, and norepinephrine, and gamma-aminobutyric acid (43). B vitamins may therefore affect central metabolism, brain function, and the modulation of mood, given their role in neurotransmitter production (44–47). A lack of basic B vitamins (folic acid, B6, and B12) in the diet is also proposed to impact on the rate of brain atrophy associated with mild cognitive impairment (MCI) and with healthy aging (15).

**Figure 1:**
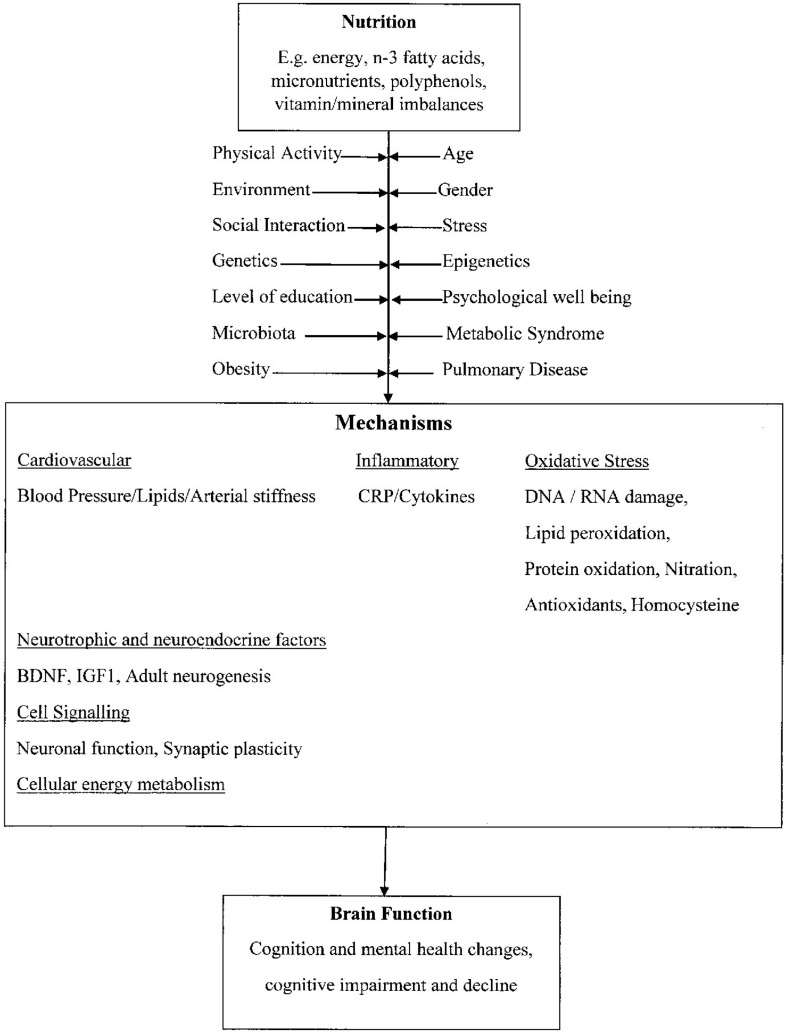
**Proposed mechanisms that link nutrition to changes in brain function**. Adapted from Dauncey (23), with permission to reprint from Cambridge Press and authority to alter.

It has also been proposed that a high intake of seafood and other sources of long-chain omega-3 polyunsaturated fats (LC-n3-FA) may have long-term beneficial effects on cognitive function (43, 48, 49). For example, energy balance and LC-n3-FA act *via* BDNF, and insulin-like growth factor-1 (IGF-1) can alter the expression of a number of protein pathways that are involved in neuronal function, plasticity, and neurogenesis (50). The addition of regular fish consumption in one’s diet has also been shown to maintain gray matter volumes in the hippocampus, precuneus, posterior cingulate, and orbital cortex (51). As shown in Figure 1, these are examples of the many factors that can potentially modulate the action of nutrition on brain function.

## The Mediterranean Diet

The MedDiet was initially investigated in studies that described the food consumption in countries in Southern Europe. In particular, the dietary intake of the Mediterranean countries of Italy, Yugoslavia, and Greece were investigated, as these populations demonstrated a higher than average life expectancy, lower rates of cardiovascular disease, cancer, and other chronic disorders (52–54). The key components of a MedDiet are abundant consumption of plant foods, such as leafy greens, fresh fruit and vegetables, cereals, beans, seeds, nuts, and legumes. The MedDiet is also low in dairy, has minimal red meats, and uses olive oil as its major source of fat (14).

Leafy vegetables are an important source of folate and other B vitamins (55, 56). The MedDiet has been reported to be protective against diseases associated with chronic inflammation, cancer, diabetes, obesity, pulmonary disease, cardiovascular disease, and cognitive disorders (57). A diet with the nutritional qualities of the MedDiet has been shown to reduce homocysteine levels, considered a risk factor for age-associated cognitive decline (58, 59). The MedDiet pattern is largely void of refined sugar, cholesterol, and trans fats, aspects of diet that are considered to be associated with poor cognitive outcomes in older age (60, 61); with saturated fats impacting negatively on learning and memory and the potential for increasing metabolic distress (62). The effects of poor diet may be overcome by changing nutritional consumption, suggesting that a diet high in fruit and vegetables may assist in improving brain functioning over time (4, 43, 63–66).

There have been a substantial number of population/epidemiological studies that have investigated adherence to a MedDiet and health outcomes, such as the population of 22,043 adults in Greece who completed extensive food frequency questionnaires (FFQs) (26). Although this study did not review the effect of the MedDiet on cognition, it did indicate that adherence resulted in reduced mortality after 44 months of follow-up (26). Adherence to the MedDiet was also found to reduce the risk of myocardial infarction (27). The Italian Longitudinal Study on Aging, which was designed to assess the prevalence and incidence of certain chronic conditions in an older population, had a sample size of 5,632 subjects aged 65–68 years and found that an intake of 46 g per day or more of monounsaturated fatty acids, primarily from olive oil, as a part of the basic MedDiet, appeared to be protective against age-related changes in cognitive function (67). In the Chicago Health and Aging Project (CHAPS) longitudinal study that assessed the incidence of AD and the habitual diet of 6,158 participants, it was found that greater adherence to all three healthy dietary styles that were assessed, including the MedDiet, was associated with a reduced incidence of AD. In a cross-sectional analysis of an Australian study, it was found that MedDiet adherence was not related to cognitive function. However, the consumption of plant foods associated with the MedDiet had a positive relationship with improving physical function and general health, while being negatively associated with anxiety, depression, and perceived stress (68).

These studies have provided evidence that the MedDiet may be protective against cognitive decline. However, there are potential confounding issues associated with both cross-sectional and longitudinal population studies that may not take into account minor reactive and biological changes that cannot be identified by observation.

## The Mediterranean Diet and Cognition – Evidence from Longitudinal and Prospective Trials?

Over the past 15 years, there has been substantial research into the impact of adherence to the MedDiet pattern with respect to overall cardiovascular health, cognitive health, and the potential to reduce the onset of dementia and AD. The understanding of what a MedDiet is and the potential biological markers and nutritional requirements of this diet have also been investigated. It is also important to segment the studies with clinical interventions from those that are considered observational studies.

This review focuses on studies that evaluate longitudinal and prospective trials in accordance with the specified criteria. The review explores our understanding of how a MedDiet may impact cognitive processes to gain an understanding of the cognitive endpoints following adherence to a MedDiet. Moreover, this review examines the various methodologies by which the MedDiet has been assessed and the assessment tools used to evaluate cognitive outcomes as well as identifying the key cognitive domains that that have been shown to be altered by adherence to the MedDiet.

## Methods

### Study Selection

The following key words were searched: cognition, cognitive measures, control group, non-interventional group, Mediterranean diet patterns, and Mediterranean diet. In addition to these key words, for inclusion, the following conditions had to be met: the study must be a randomized or non-randomized trial, longitudinal, or a prospective cohort study.

Articles were excluded if they were focused on dementia only, depression only, child or adolescent depression only, AD only, Parkinson’s disease only, stroke or cardiovascular disease only, diabetes only, or a review of published work.

A literature search was carried out with the focus on articles published between the years from 2000 to 2015. The following search engines were used: Directory of Open Access Journals, EBSCO Host Academic Search Complete Gale Cengage Academic One File, Google Scholar, Health Reference Center Academic, Health Reference Center (Gale), Highway Free Press, Journals @ Ovid, Journal reports, Medline, PubMed, Science Direct Freedom Collection, Scopus, Springer Link, Web of Science, Wiley Online Library, and Taylor & Frances Journal Complete.

The data extraction was carried out by one researcher, who completed the assessment in cooperation with the other authors to ensure that the capture of the information was relevant and consistent with the selection and exclusion criteria.

The data extracted from each article included (1) characteristics of the trial participants (number of participants, gender, and age); (2) study title, with respect to relevance and terminology used; (3) year of publication; (4) study design and country of origin; (5) sample size; (6) FFQ; (7) MedDiet assessment; (8) cognitive assessments used; (9) cognitive sub-measures; and (10) study outcomes.

A study was included in the review if it had a measure of a MedDiet, a Mediterranean Diet Score (MedDietS) based on a numeric point scale as a result of any form of food frequency assessment, or total food intake. In addition, studies were also included if they used a MedDiet intervention with other key dietary components.

## Results

### Included Studies

**Figure 2:**
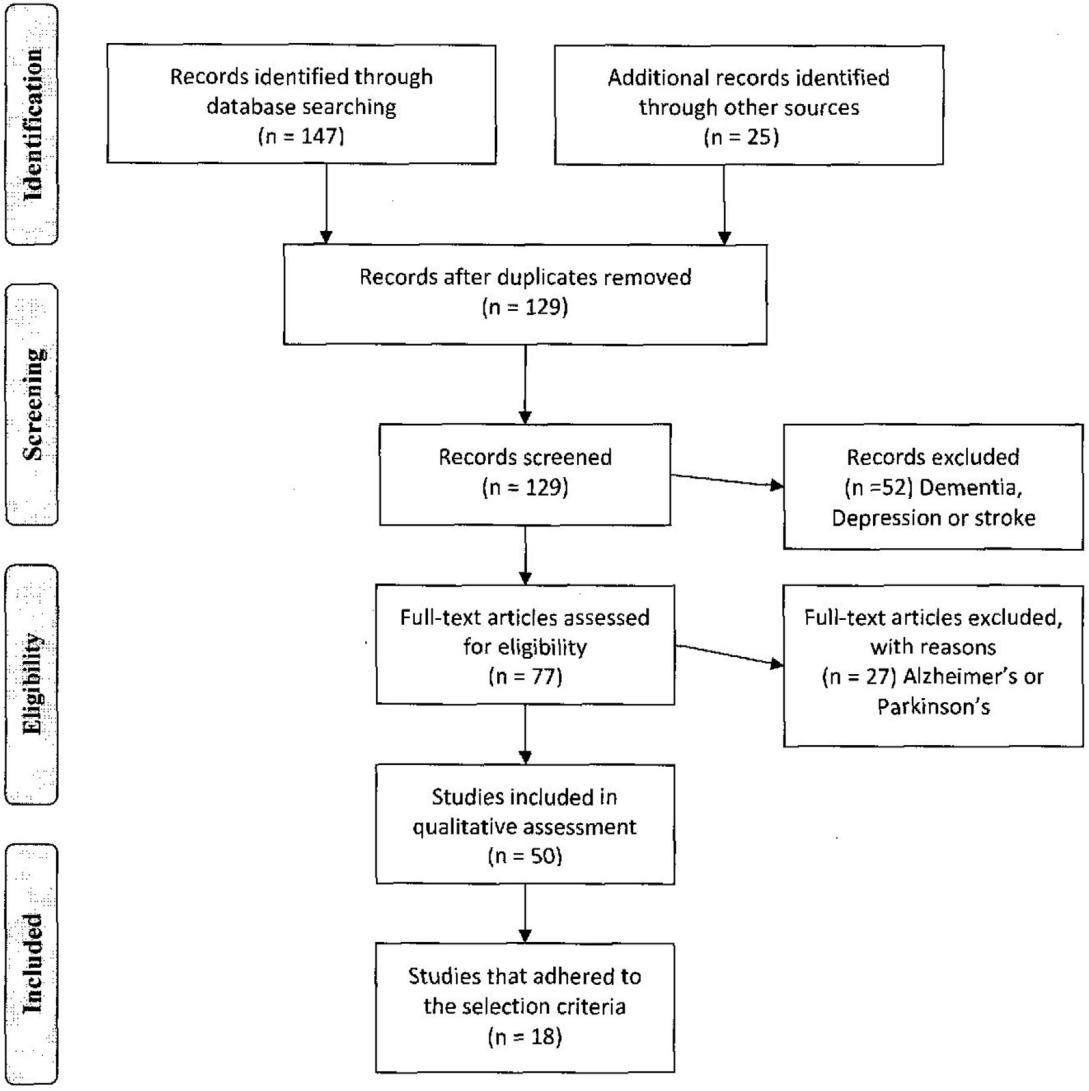
**PRISMA diagram of the study selection process**.

Independent searches utilizing the selection criteria identified 173 trials, and after adjustment for duplicates, 129 articles remained for assessment. Of these, 79 were discarded after reviewing the abstracts and article content as they did not meet the criteria. This left the full text of 50 articles for general review. After allowing for the inclusion criteria and more extensive examination, 18 studies met the inclusion criteria. The selection procedure and the total number of eligible trials reviewed are shown in Figure 2.

Table 1 shows the data extracted from each of the trials. In summary, the age range of the participants that were included in this selection varied considerably, with two studies including younger adults (19–40 years), three studies looking at middle age (45–65 years), nine in the range (65–75 years), and four in the range (75+ years).

**Table 1:** **Summary of the included studies**.

Study no.	Study author	Study design (Country of origin)	Age (years)	*N*	Food frequency assessment	MedDiet assessment	Cognitive assessment	Cognitive sub-measures	Outcome
1	Scarmeas et al. (69)	Prospective cohort (USA)	76+	1,984	61-item FFQ. Willett et al. (70)	Scored as in Trichopoulou et al. (26)	Visual Retention Test, Weschler Adult Intelligence Scale (WAIS)	Storage and retention, disordered memory and learning, visual retention	Higher adherence to a MedDiet decreases risk of AD
2	Féart et al. (71)	Prospective cohort (France)	65+	1,410	FFQ and 24-h dietary recall	Scored as in Trichopoulou et al. (26)	MMSE, Isaac Set Test, Benton Visual Retention Test, Free and Cued Selective Reminding Test	Orientation, registration, attention, calculation, recall and language, controlled attention memory, semi-verbal fluency, speed of verbal production	MedDiet slower MMSE cognitive decline and not risk for incident dementia
3	Scarmeas et al. (72)	Multiethnic community longitudinal study (USA)	76–78	1,393	FFQ. Willett (70)	Scored as in Trichopoulou et al. (26)	Neuropsychological battery, Global Clinical Dementia Rating (CDR)	Long- and short-term memory, judgment, and problem-solving	Higher adherence to a MedDiet associated with reduced risk of MCI, reduced conversion of MCI to AD
4	Gu et al. (73)	Prospective cohort (USA)	65+	1,219	Semi-qualitative 61-item FFQ. Willett (70)	Scored as in Scarmeas et al. (72) and Trichopoulou et al. (26)	15 neuropsychological tests	Long- and short-term verbal memory, short-term non-verbal memory, orientation, construction, abstract reasoning, language	Higher adherence to a MedDiet associated with reduced risk of AD
5	McMillan et al. (74)	Randomized single-blind parallel groups (Australia)	19–30	27	FD	None, MedDiet intervention with 93% adherence	COMPASS	Attention, working memory, long-term working memory, executive function, response time, word recognition	MedDiet increases vigor, alertness, contentment, changes in cognitive function
6	Tangney et al. (34)	Data analysis of the CHAP study Longitudinal study (USA)	≥65	3,790	Harvard FFQ	Scored as in Panagiotakos et al. (75)	MMSE, East Boston tests of immediate and delayed recall, symbols digit modalities test	Immediate and delayed recall, orientation, registration, attention, calculation, recall and language	MedDiet associated with reduced rate of cognitive decline with older age
7	Cherbuin and Anstey (35)	Longitudinal study (Australia)	60–64	1,528	CSIRO FFQ	Scored as in Scarmeas (72) and Trichopoulou (26)	International consensus criteria, CDR	Perceptual memory, motor function, simple and complex reaction time	MedDiet not protective against cognitive decline
8	Vercambre et al. (76)	Prospective cohort (women only) (USA)	65+	2,504	FFQ 116 food groups. Willet (70)	Scored as in Trichopoulou (26)	TICS, telephone adaptation of the MMSE, verbal memory with the TICS 10-word list, East Boston memory test (EBMT)	Immediate and delayed recall, orientation, registration, attention, calculation, recall, and language	No association between MedDiet and 5-year cognitive change
9	Kesse-Guyot et al. (62)	Prospective cohort (France)	65.4 ± 4.6	3,083	French food composition table, computerized questionnaire FFQ	Mediterranean Dietary Pattern Score. Rumawas et al. (77)	Rappel indices 48-item (cued recall), verbal fluency tests of lexical/semantic memory	Episodic memory, working memory, verbal fluency, phonometric fluency	MedDiet adherence was not associated with cognitive performance overall
10	Martinez-Lapiscina et al. (78)	Multicenter randomized (Spain)	74.6 ± 5.7	522	Semi-quantitative FFQ. Fernández-Ballarth et al. (79)	14-item questionnaire. Martínez-gonzález et al. (27)	MMSE and Clock-Drawing Test (CDT), CDT language and comprehension	Working memory, visual and spatial orientation, orientation, registration, attention, calculation, recall and language	Higher adherence to a MedDiet intervention improved cognition compared with a low-fat diet
11	Samieri et al. (80)	Prospective epidemiological (USA)	≥70	16,058	USDA FFQ	Scored as in Trichopoulou (26)	TICS, immediate and delayed recall of the EBMT, delayed recall of the TICS 10-word list, category fluency, Digital Spin backward	Global cognitive scores, immediate and delayed recall, verbal memory, language	Long-term adherence to the MedDiet pattern is modestly associated with global cognitive function and verbal memory in later life, but not with a cognitive change after a 6-year period
12	Samieri et al. (81)	Randomized double-blind (USA)	65+	6,174	131-item semi-quantitative FFQ. Rimm et al. (82)	Scored as in Fung et al. (83)	TICS, immediate and delayed recall of the EBMT, delayed recall of the TICS 10-word test, category fluency	Recall memory, working memory, immediate and delayed recall, language	MedDiet not associated with cognitive decline; though certain dietary components may be related and warrant further investigation
13	Titova et al. (84)	Prospective cohort (Sweden)	70	194	FFQ. Rimm (82)	Scored as in Trichopoulou (26)	Swedish 7-min screen for cognitive decline and dementia, Benton temporal orientation, CDT, categorical verbal fluency	Recall memory, temporal orientation, attention, verbal fluency	Reduced intake of meat and meat products associated with greater brain volume and better cognitive performance
14	Tsivgoulis et al. (85)	Prospective cohort (USA)	45–90	17,478	Food intake *via* a 1-week diary	Scored as in Scarmeas et al. (72) and Féart et al. (71)	6-item screen. Callahan et al. (86)	Delayed memory	High adherence to a MedDiet associated with reduced rate of incident cognitive impairment
15	Ye et al. (87)	Longitudinal cohort (Puerto Rican adults in USA)	45–75	1,269	NHNS FFQ	Scored as in Trichopoulou (26)	MMSE, word learning list forward and back, digit spin Stroop test, verbal fluency	Memory, attention, orientation, registration, calculation, recall and language learning, immediate recall, recognition and percent recognition	High adherence to a MedDiet associated with greater cognitive function and reduced risk of cognitive impairment
16	Galbete et al. (88)	Prospective cohort (Spain)	62 ± 6	824	Validated semi-quantitative FFQ, MyPyramis Equivalent Database 2.0 for USDA Survey Foods 2003–2004. Bowman et al. (89)	Scored as in Trichopoulou (26)	Spanish TICS	Immediate memory, delayed recall, orientation, attention, calculation, language	Higher adherence to a MedDiet associated with better cognitive outcomes
17	Lee et al. (90)	Randomized trial – balanced crossover groups (Australia)	20–38	24	FD	None, ≥80% adherence for dietary intervention	COMPASS	Attention, working memory, long-term memory, executive function	A MedDiet potential to enhance mood, cognition, and cardiovascular health in young adults
18	Valls-Pedret et al. (91)	Randomized trial – parallel groups (Spain)	67 (mean)	447	MedDiet program	None/high compliance	MMSE, Rey Auditory Verbal Learning Test, Animals Semantic Fluency, Digit Span from the WAIS, Verbal Paired Association	Memory, attention, executive function	A MedDiet is associated with improved cognitive function

*AD, Alzheimer’s disease; FD, food diary; FFQ, Food Frequency Questionnaire; CDR, global clinical dementia rating; CDT, clock-drawing test; CHAP, Chicago Health and Aging Project; COMPASS, computerized mental performance assessment system; CSIRO, Commonwealth Scientific and Industrial Research Organisation; EBMT, East Boston memory test; MedDiet, Mediterranean diet; MCI, mild cognitive impairment; MMSE, mini–mental state examination; TICS, telephone interview for cognitive status; USA, United States of America; USDA, United States Department of Agriculture; WAIS, Wechsler Adult Intelligence Scale*.

These trials including a number of different study designs are presented in Table 1.

### Nutritional Intake Assessments

One of the key elements required to assess the adherence to the MedDiet is to gain an understanding of the volume of food intake and the particular food groups used for assessment. The common approach to achieve this outcome is to have participants complete a food diary (FD) or an assessment of their food consumption through the use of a FFQ at time points as part of the trial protocol. In all of the original research studies that met the selection criteria for this analysis, the participants had completed either an FFQ or an FD. Of the 18 studies identified, 14 utilized an FFQ, 3 utilized an FD, and 1 had a food program. The total number of categories of foods varied from a limited 20 groups through to 250 different food groups, with data being collected over a range of time frames.

### Mediterranean Diet Scores

The assessment of the MedDietS has been discussed in the literature since 2003. This initial research identified 150 food groups and placed these into 14 inclusive food groups (26). A sex-specific median calculation was used to determine the baseline value to assess the MedDietS with the 14 inclusive food groups, or nutrients were considered, where some groups were combined to eventually calculate a score ranging from 0 to 9. The sex-specific median allows for a comparative cut off to be made between genders on food consumption (26). Beneficial foods, such as vegetables, legumes, fruits, nuts, cereals, and fish, were assigned a value of 0, if a person’s consumption was below the median, and a score of 1, if it was above the median. With food components considered to be detrimental to health, such as meat, poultry, and dairy, consumption above the median was scored as 0, and intake below the median was scored as 1. For alcohol, a score of 1 was given, provided consumption was within a specified range. When considering fat intake, the ratio of monounsaturated lipids to polyunsaturated lipids was evaluated with a higher ratio being more acceptable and a score of allocated accordingly.

Of the research included in this review, 10 papers referenced the Trichopoulou et al. (26) study to calculate a MedDietS, while 5 created a MedDietS utilizing a mean score. There are three papers that did not produce a MedDietS. When making an assessment of the MedDietS, 6 studies used mean scores, 11 used median scores, and 1 used a regression residual method. Three studies used a direct or total food amount. Although many studies utilized the original method to analyze the data, only four followed the sex-specific median approach. These were Articles 1, 3, 4, and 9 in Table 1.

### Cognitive Assessments

Generally, cognitive assessments utilize the mini–mental state examination (MMSE) (92). Of the 18 original research papers shown in Table 1, 6 utilized this tool in combination with other tests. Another seven studies used cognitive assessments administered as part of neuropsychological test batteries and other scales. These included the East Boston tests of immediate and delayed recall, the clinical dementia rating scale, 6-item screeners, telephone interviews, category fluency, Stroop tests, clock-drawing, and others. All of which were used as paper-based assessments. Two studies used a computer-based assessment for cognition – the computerized mental performance assessment (COMPASS) (74, 90). The cognitive domains that were assessed included measures of attention, working memory, long-term memory, and executive function.

### Cognitive Results

Due to the heterogeneity of the selected studies that included a variety of experimental designs, cohorts, sampling times, as well as very diverse dietary and cognitive measures, it was not possible to pool data sets (102, 103). Instead, we used the dietary assessment approach as a framework to report the cognitive outcomes from each study and discuss consistencies/inconsistencies more qualitatively.

Of the research that completed their assessment using a mean score to determine their MedDietS (Studies 6, 10, and 11), two found that higher adherence to the MedDiet resulted in slow rate of cognitive decline or improvements in cognition, including memory and immediate and delayed recall.

For those studies that used a median score for their MedDietS (Studies 2, 7, 8, 12, 13, 14, and 16), three showed that adherence to the MedDiet improved cognitive scores utilizing assessments of recognition memory, delayed recognition memory, or that this diet reduced the rate of cognitive decline utilizing assessments of working memory. In one study (Study 12), no relationship was found between the MedDiet intervention and cognitive decline; however, the authors did suggest that a specific food category, whole grains, merits further investigation with regard to cognition (81).

Four of the studies (Studies 1, 3, 4, and 9) also used a median score (sex-derived) to calculate the MedDietS. These studies analyzed the median score separately for men and women, as was done in the original study by Trichopoulou et al. (26). In three of these studies (Studies 1, 3, and 4), it was demonstrated that a higher adherence to a MedDiet was associated with a trend to reducing the risk of developing MCI (72), as measured using a global assessment and memory cognitive domains.

One study that did not use a mean or median calculation used a regression residual analysis method (Study 15). In this study, it was concluded that greater adherence to a MedDiet was associated with better cognitive function and a strong correlation with improvement in cognitive reserves when using MMSE, relating these outcomes to the cognitive domains of memory and attention (87).

In three studies, the absolute amount of food consumed in each category was used to assess adherence to a MedDiet. All reported high adherence to a MedDiet (Studies 5, 17, and 18). These studies demonstrated that a MedDiet supplemented with olive oil or nuts was associated with improved cognitive function and also that the MedDiet had the potential to enhance aspects of mood and cardiovascular function (74, 90, 91).

Finally, these trials were conducted in countries around the world with positive outcomes demonstrated both within and outside the Mediterranean region. There were six studies reporting positive cognitive outcomes in the USA, one study in France, three in Spain, one study in Sweden, and two in Australia.

## Discussion

This review has explored the contention that the MedDiet has a long-term positive effect on cognitive function. The results of the dietary pattern, cognitive decline, and dementia reviews also support the proposition that adherence to a MedDiet pattern is associated with less cognitive decline, dementia, or AD [69]. The primary aim of this review was to determine if adherence to a MedDiet produces favorable outcomes in relation to improved cognition or minimization of cognitive decline. Of the 18 research articles examined, 13 demonstrated that higher adherence to a MedDiet was related to either slowing the rate of cognitive decline, minimizing the conversion to AD or improving the cognitive function. Five of the 18 studies did not demonstrate that MedDiet adherence had a protective effect against cognitive decline or did not improve cognition. Irrespective of the study design, the studies that demonstrated improved cognitive outcomes examined the broad cognitive domains of attention, memory, and language. The studies that focused on the cognitive domains of motor or action did not report favorable outcomes when related to MedDiet adherence. The more specific cognitive domains that improved with MedDietS were memory, delayed recognition, executive function, long-term working memory, and visual constructs. These are mainly tests of fluid intelligence that decline with age (7). Interestingly, benefits to cognition afforded by the MedDiet were not exclusively in older individuals. The two studies included that were in younger adults both found improvements in cognition using computerized assessments.

It is noted that although there are several different designs within the studies examined, including randomized or non-randomized, that were longitudinal or prospective, and that may or may not have been interventional, it was difficult to assess if one or more particular designs reported more favorable outcomes with respect to adherence to a MedDiet and cognitive outcomes. It was also difficult to assess whether or not the calculation of the MedDietS, based on the original analysis of Trichopoulou et al. (26), yielded more favorable cognitive outcomes. More specific approaches utilizing a median, mean, or sex-derived determination of MedDietS were similarly difficult to conclude if one approach was better than the other with respect to cognitive outcomes, given the small number of studies and the mixed approaches for assessing cognition.

Six studies used the MMSE to examine a global measure of cognition. This measure was used mainly in trials examining cognitive decline and conversion to AD over a longer period of time. This seems reasonable given that the MMSE is a course tool for measuring cognition and is recommended mainly to identify accelerated aging and neurocognitive disorders, such as MCI and AD (93). Of the trials that used the MMSE to assess cognitive decline, three of the six trials showed either reduced cognitive decline or reduced conversion to MCI or AD. MMSE was also used to assess cognitive improvement – three out of six trials showed improvement in relation to adherence to a MedDietS. A higher rate of positive cognitive outcomes may have been realized if a more sensitive cognitive assessment tool was used. The studies that used the MMSE as their primary source of cognitive assessment measured cognitive decline as compared with the computer-based assessments that focused on cognitive improvement. The majority of the studies that used the MMSE also included cognitive assessments that measured memory, attention, language, and mood, such as the COMPASS, delayed recall from the Boston memory tests, the telephone interview of cognitive status (TICS) 10-word list, Stroop tests, verbal fluency measures, lexical semantic memory assessments, and clock-drawing tests. Only two of the trials used a computer-based assessment for cognition, the COMPASS, both showing evidence of improved cognitive functioning in a short-term study with a 10-day intervention. The non-computer-based neuropsychological assessments in general showed improved cognition or a reduced cognitive decline in relation to a MedDietS. There were 10 studies using these other neuropsychological assessments with 6 showing cognitive change with regard to a MedDietS.

The studies examined used a range of methods to derive a MedDietS. Some studies used an FD, while others used various FFQ assessments. An FFQ is a way to estimate the food consumed over an extended period 6–12 months. An FD is a more specific and detailed assessment conducted over a number of days to quantify what one actually eats. There is no standardized pattern or agreed types of foods included in these forms of dietary assessments. The foods consumed can be considered specific to a region or country of origin, and the foods vary between studies, as they have been conducted in different countries and geographic areas. Generally, the food intake is recorded as it is relevant to the specific cohort under investigation. The FFQ has been found to be reproducible and have good comparative validity. The FFQ has demonstrated dietary intervention effects to be relatively accurate (94–96). When converted to different languages, the validity of the FFQ was also reproducible, such as with the Brazilian, Chinese, and Italian populations (97–100).

Across the 18 studies, there was no standardization of the most appropriate FFQ. There was a large variation in the FFQs adopted, and the selection of food groups ranged from 70 to 250 different groups. It is also important to consider portion sizes, as some studies used food frequency alone. It is more appropriate for comparison to include portion size by food groups with respect to gender and ethnicity (101). Portion size needs to be determined based on the norms for the country under investigation and reported in grams per day for equivalence. The adherence to a standardized pattern of a MedDiet as demonstrated in the initial research conducted in 2003, Trichopoulou et al. (26) also needs to be assessed. This original research, which forms the focus of many of the studies conducted to date, used 14 all-inclusive food groups that were evaluated in grams per day, and consequently prospective studies need to adhere to this specific pattern of evaluation to ensure valid comparisons can be made.

To ensure that direct correlations can be made between studies, it would be of value to have a standardized computer-based FFQ that is easy to administer and simple to review. The use of a standardized FFQ that utilizes 14 all-inclusive food groups all evaluated to grams per day, and this would enable more accurate portions of food taken (grams per day) to be evaluated to obtain a MedDietS.

Interestingly, positive cognitive effects were found in countries around the world, including those outside of the Mediterranean region. Many of these positive studies used an approach where a mean or median is determined for each food category, and adherence for the individual is scored relative to this mean or median. In this way, habitual dietary habits are normalized with respect to the local region. Change over time is assessed relative to baseline scores to assess whether or not greater adherence supports better cognitive health. While this makes it difficult to compare absolute measures of dietary adherence in relation to a more traditional Mediterranean diet (26), it is encouraging that measurable shifts in adherence within a local population are effective in improving cognition or retarding cognitive decline. An important challenge will be to demonstrate a sustainable long-term shift in diet within a population that also confers cognitive benefits.

## Conclusion and Future Directions

This review has analyzed longitudinal and prospective trials in accordance with the specified criteria to gain an understanding of how a MedDiet may impact cognitive processes. The level of adherence to the MedDiet has subsequently been evaluated against various cognitive domains to ascertain the potential protective nature of this diet pattern with respect to minimizing cognitive decline, improving cognitive decline, or reducing the incidence of conversion of MCI to AD.

The overall outcome from this review indicates that there is encouraging evidence that a higher adherence to a MedDiet is associated with improving cognition, slowing cognitive decline, or reducing the conversion to AD.

With few studies available to assess benefits of a MedDiet intervention in a healthy older population, it is recommended that further randomized controlled trials need to be conducted. These trials need to include a standard food consumption assessment that can be validated to determine the impact of cognitive changes over time. It is also recommended that future RCTs utilize validated computer-based cognitive batteries that are sensitive to cognitive faculties, which are compromised with age and potentially amenable to interventions, such as a MedDiet. Future studies should also consider the use of blood, cardio, and fecal biomarkers that will allow for mechanisms of action to be evaluated. Biomarkers will provide more direct measures of dietary status and will potentially elucidate important biological changes that relate to brain function and modifiable risk factors.

Further focused research in this area is important due to the expected extensive population aging over the next 20–30 years. The utilization of such a dietary pattern will be essential as part of the armamentarium to maintain quality of life and reduce the potential social and economic burden of dementia.

## Author Contributions

There have been substantial contributions to the conception and design of this review by all authors, and all authors have been involved in revising the work critically for important intellectual content. The final approval of the version to be published has been agreed by all authors, and an agreement to be accountable for all aspects of the work in ensuring that questions related to the accuracy or integrity of any part of the work has been appropriately investigated and resolved.

## Conflict of Interest Statement

The authors declare that the research was conducted in the absence of any commercial or financial relationships that could be construed as a potential conflict of interest.
